# Immediate effects of joint- versus soft-tissue-oriented manual therapy on lower-limb function and muscle mechanics in individuals with flexible pes planus: a randomized controlled trial

**DOI:** 10.1007/s11845-026-04464-2

**Published:** 2026-05-26

**Authors:** Bensu Sogut-Kazan, Ezgi Unuvar-Yuksel, Hande Guney-Deniz

**Affiliations:** 1 Department of Sports Physiotherapy and Rehabilitation, Faculty of Physical Therapy and Rehabilitation, Hacettepe University, Ankara, 06100 Türkiye; 2 Department of Physiotherapy and Rehabilitation, Faculty of Health Sciences, Izmir Demokrasi University, Izmir, Türkiye; 3 Department of Musculoskeletal Physiotherapy and Rehabilitation, Faculty of Physical Therapy and Rehabilitation, Hacettepe University, Ankara, Türkiye

**Keywords:** Balance, Flatfoot, Mobilization, Myofascial release, Myotonometry

## Abstract

**Background:**

Flexible pes planus (FPP) is associated with altered foot biomechanics, reduced ankle dorsiflexion, and impaired neuromuscular function, which may affect balance and performance. Manual therapy targeting joint and soft tissue structures is commonly used; however, the comparative immediate effects of these approaches remain unclear.

**Aims:**

To compare the immediate effects of joint-oriented manual therapy (J-MT) and soft-tissue–oriented manual therapy (ST-MT) on dorsiflexion range of motion (DFROM), foot morphology, dynamic balance, and muscle mechanical properties in physically active adults with FPP.

**Methods:**

In this prospective randomized controlled trial, 45 adults (18–35 years) with FPP were allocated to J-MT (*n* = 15), ST-MT (*n* = 15), or control (*n* = 15) using concealed block randomization. J-MT involved ankle–foot joint mobilization, while ST-MT targeted the triceps surae, Achilles tendon, and plantar fascia. Controls received no intervention. The primary outcome was DFROM (weight-bearing lunge test). Secondary outcomes included navicular drop (ND), Foot Posture Index (FPI), Y-Balance Test, and myotonometric muscle properties. Assessments were performed pre- and post-intervention.

**Results:**

Both J-MT and ST-MT significantly reduced ND and FPI versus controls (*p* < 0.05), with no between-group difference. J-MT improved DFROM and anterior reach more than ST-MT (*p* < 0.05). No significant differences in dynamic balance were observed. ST-MT reduced soleus tone and tibialis anterior stiffness, while both interventions improved soleus elasticity (*p* < 0.05). No adverse events occurred.

**Conclusions:**

J-MT and ST-MT produce immediate, parameter-specific effects in FPP. J-MT enhances dorsiflexion, whereas ST-MT better modulates muscle mechanical properties, supporting the selection of targeted interventions.

**Supplementary Information:**

The online version contains supplementary material available at https://doi.org/10.1007/s11845-026-04464-2.

## Introduction

Flexible pes planus (FPP) is characterized by the collapse of the medial longitudinal arch during weight-bearing, accompanied by excessive pronation and rearfoot eversion, which alters lower-limb biomechanics [1, 2]. This altered alignment has been associated with injuries such as plantar fasciitis, Achilles tendinopathy, and midfoot instability, as well as lower extremity kinetic chain deviations including knee valgus, hip internal rotation and alterations in gait pattern [3, 4]. Although FPP is not always symptomatic, its potential to cause functional limitations and overuse injuries highlights the need for targeted interventions.

FPP can impair dynamic balance and ankle dorsiflexion range of motion (DFROM) [5] due to changes in foot biomechanics, shortened calf muscles, and reduced proprioception [6, 7]. Altered soft tissue properties, including increased stiffness and tone of the Achilles tendon [8] and lower-leg muscles [9], may further compromise shock absorption and functional performance. Despite the frequent clinical use of manual therapy to address these impairments, the relative contributions of different manual therapy approaches remain unclear.

Manual therapy approaches are frequently used in the management of FPP [10–12]. Soft-tissue–oriented manual therapy (ST-MT), such as myofascial release, targets fascial restrictions to restore mobility and reduce pain [13], while joint-oriented manual therapy (J-MT), such as mobilization, aims to enhance joint mobility and proprioception while reducing pain [14, 15]. Both approaches have shown benefits for dorsiflexion, pain, and balance; however, their comparative effects in individuals with FPP remain unclear [16–18].

To date, no randomized controlled trial has directly compared the immediate effects of ST-MT and J-MT using objective measures such as myotonometry and dynamic balance testing. Therefore, the primary aim of this randomized controlled trial was to compare the immediate effects of J-MT and ST-MT on ankle dorsiflexion range of motion in physically active adults with asymptomatic flexible pes planus. Secondary aims were to evaluate their effects on foot morphology, dynamic balance and passive mechanical properties measured via myotonometry. We hypothesized that both approaches would increase dorsiflexion range of motion, while also improving foot morphology, dynamic balance, and reducing tissue stiffness. The findings of this research may contribute to a better understanding of the differential efficacy of manual therapy interventions in asymptomatic flexible pes planus.

## Methods

### Study design and ethical approval

This study was a prospective, three-arm, parallel-group randomized controlled trial conducted in accordance with the Declaration of Helsinki and reported in accordance with the CONSORT 2025 guidelines. The protocol was approved by Institutional Ethics Committee of the authors’ affiliated university (Approval No: FTREK25/139) and prospectively registered at ClinicalTrials.gov (NCT07326020; registered November 29, 2025). Participant recruitment and baseline assessments were initiated after trial registration.

### Participants

Forty-five physically active volunteers aged 18–35 years were recruited via social media advertisements. All participants provided written informed consent before participation. Assessments were performed at the laboratory of the authors’ affiliated university. Participants had flexible pes planus, defined as a navicular drop (ND) ≥ 10 mm and a Foot Posture Index (FPI) score > 6. All participants had Tegner Activity Scale scores between 4 and 6. Exclusion criteria included lower extremity surgery or trauma, professional athletic participation, BMI > 30 kg/m^2^, neurological or metabolic disorders, posterior tibial tendinopathy, any treatment for pes planus within the past year.

### Randomization, blinding and study protocol

Participants with FPP were randomly allocated to one of three groups (J-MT, ST-MT, or control; *n* = 15 per group) using a computer-generated block randomization sequence (a block size of 6) created by an independent researcher not involved in assessments or interventions (HGD). Allocation concealment was ensured using sequentially numbered, opaque, sealed envelopes opened only after baseline assessment. Outcome assessors and data analysts were blinded to group allocation. Participants and therapists were not blinded due to the nature of the interventions.

Each participant attended a single laboratory session. Participants received no concomitant care or medications (e.g., analgesics) during the study, and were instructed to avoid strenuous exercise for 24 h prior to the session. Baseline assessments were performed on the more affected limb, determined based on greater navicular drop values.

### Intervention protocol

Participants assigned to intervention groups received the allocated manual therapy after a 15-min rest period following the baseline assessments. Post-intervention measurements were performed within 5 min after intervention. Participants in the control group received no intervention and completed post-assessments after a 15-min rest period to control for time effects. All interventions were delivered by the same senior physiotherapist (BSK) with 8 years of clinical experience following standardized procedures. After completion of all post-intervention assessments, all participants were provided with general recommendations for the management of flexible pes planus, including corrective exercise programs and orthotic support when appropriate.

#### Soft-tissue–oriented manual therapy group

Participants allocated to the ST-MT group received 15 min of soft-tissue–oriented manual therapy, including myofascial release techniques applied to the gastrocnemius, soleus, Achilles tendon, and plantar fascia following the protocol by Ajimsha et al. [13]. The therapist applied low-load, long-duration pressure synchronized with the participant’s active movement. Detailed intervention protocols are provided in Online Resource 1.

#### Joint-oriented manual therapy group

Participants received joint mobilization techniques targeting the ankle–foot complex, following the protocol described by Shashua et al. [19]. Mobilizations included anterior–posterior talocrural joint glides in non–weight-bearing and weight-bearing positions, subtalar mobilization for eversion/inversion, and midtarsal mobilization for pronation/supination. Detailed intervention protocols are provided in Online Resource 1. Each session lasted 15 min.

#### Control group

Control participants underwent baseline and post-rest assessments identical to the intervention groups but did not receive any intervention or sham treatment. The 15-min rest period controlled for time-related effects.

### Outcome measures and assessment procedures

The primary outcome was ankle dorsiflexion range of motion assessed using the weight-bearing lunge test. Secondary outcomes included foot morphology, dynamic balance, and myotonometric measures of muscle tone, stiffness, and elasticity. Harms were assessed non-systematically by recording any adverse events reported by participants during the study period.

#### Foot morphology

The structural characteristics of the foot were assessed as outcome measures and to characterize foot structure. The navicular drop test was used to measure the vertical displacement of the navicular tuberosity from a sitting (non-weight-bearing) to a standing (weight-bearing) position, with ≥ 10 mm required for inclusion [20]. Additionally, the Foot Posture Index was utilized to evaluate multiplanar standing posture; six clinical criteria were scored from −2 to + 2, and a total score > 6 confirmed a pronated foot posture [21].

#### Ankle dorsiflexion range of motion

Ankle DFROM was measured using the weight-bearing lunge test. The maximum toe–wall distance (cm) was recorded while maintaining heel contact with the floor and knee contact with the wall [22].

#### Dynamic balance

Dynamic balance was measured using the Y-Balance Test (Move2Perform, USA) in anterior, posteromedial, and posterolateral directions [23]. Three practice trials and three recorded trials were performed in each direction. Reach distances were normalized to lower limb length (measured from the anterior superior iliac spine to the medial malleolus) using the following formula: [(reach distance/limb length) × 100]. The composite score was calculated as [(anterior + posteromedial + posterolateral reach distances)/(3 × limb length)] × 100 [23].

#### Passive mechanical properties

Muscle tone (F), stiffness (S), and elasticity (decrement, D) were measured using a Myoton-Pro device (Myoton SA, Estonia) [24]. A 3 mm device probe was applied perpendicular to the skin at standardized anatomical sites: tibialis anterior (midpoint of muscle belly), medial/lateral gastrocnemius (30% of lower leg length and 1/3 distance from fibular head, respectively), soleus (2 cm medial to midline, distal to gastrocnemius), Achilles tendon (4 cm superior to calcaneal tubercle), and plantar fascia (intersection of foot midpoint and 5th metatarsal base) [25–28]. Each variable was recorded three times, and the mean value was used for analysis. Previous research has demonstrated good reliability and construct validity of Myoton-Pro measurements for assessing passive mechanical properties of lower-extremity muscles and tendons [24].

### Statistical analysis

Sample size was determined a priori using G*Power 3.1 based on pilot data for ankle dorsiflexion range of motion measured by weight bearing lunge test, which was considered the most variable and conservative outcome measure. An effect size of 0.32, power of 0.90, and α = 0.05 indicated that at least 12 participants per group were required. To ensure adequate power, 45 participants (15 per group) were included.

All statistical analyses were performed using IBM SPSS Statistics (Version 22, IBM Corp., Armonk, NY, USA). Descriptive statistics were presented as mean ± standard deviation for continuous variables. Baseline demographic characteristics were compared among the three groups using one-way analysis of variance (ANOVA). To evaluate intervention effects over time, a two-way mixed-design analysis of variance (mixed ANOVA) was performed with group (ST-MT, J-MT, and control) as the between-subjects factor and time (pre-intervention and post-intervention) as the within-subjects factor. The group × time interaction effect was used to determine whether the change over time differed between groups. Change scores were calculated as the difference between post-intervention and pre-intervention values. For significant interaction effects, between-group comparisons were further examined using change scores, and Bonferroni-adjusted post-hoc tests were used for pairwise comparisons when significant effects were detected. Effect sizes were reported using partial eta squared (ηp^2^) for mixed ANOVA results. For pairwise comparisons, mean differences (MD) with 95% confidence intervals (CI) were reported. The level of statistical significance was set at *p* < 0.05.

## Results

**Fig. 1:**
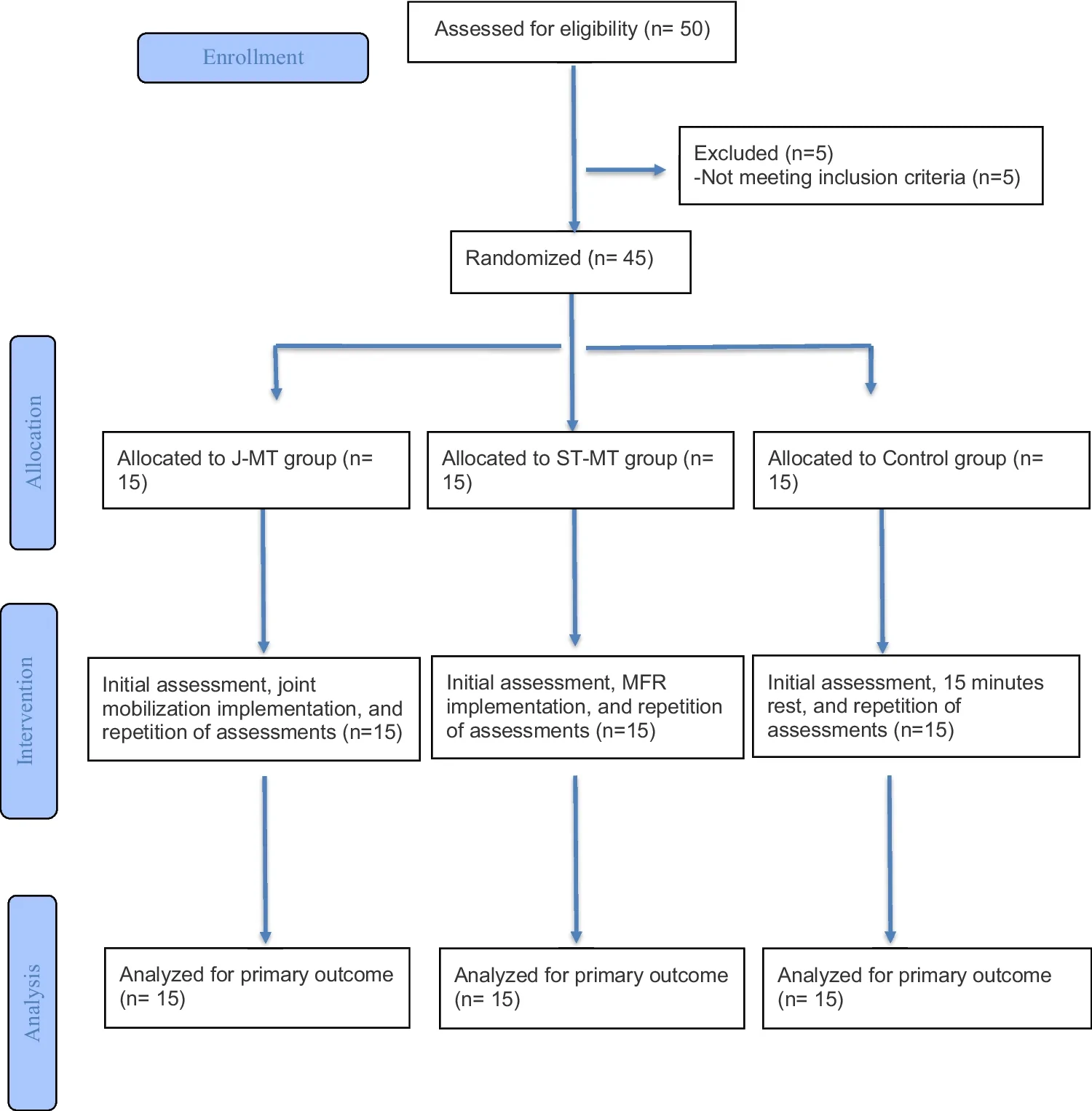
CONSORT 2025 Flow Diagram of participant recruitment, randomization, allocation to intervention groups (J-MT, ST-MT, and control), follow-up, and analysis

All 45 randomized participants completed the study and were included in the final analyses (Fig. 1). Baseline demographic and clinical characteristics were comparable among the three groups, with no significant between-group differences observed for age, height, body weight, BMI, or Tegner activity level (all *p* > 0.05) (Table 1). Baseline values of all outcome measures were comparable between the groups, with no significant differences observed in navicular drop, Foot Posture Index, weight-bearing lunge test, Y-Balance Test parameters, or myotonometric measurements (all *p* > 0.05) (Tables 2 and 4). No adverse events were reported.

**Table 1:** Demographic and baseline characteristics of participants

	ST-MT group (*n* = 15)	J-MT group (*n* = 15)	Control group (*n* = 15)	p
Age (years)	25.67 ± 5.19	25.27 ± 5.50	24.93 ± 5.42	F(2,42) = 0.070, *p* = 0.932
Height (m)	1.68 ± 0.11	1.69 ± 0.07	1.71 ± 0.09	F(2,42) = 0.694, *p* = 0.505
Weight (kg)	65.73 ± 13.87	66.87 ± 9.35	69.13 ± 11.89	F(2,42) = 0.320, *p* = 0.728
BMI (kg/m^2^)	23.11 ± 2.38	23.32 ± 2.04	23.32 ± 2.26	F(2,42) = 0.040, *p* = 0.961
Tegner activity level (score)	4.60 ± 0.82	4.93 ± 0.70	4.73 ± 0.70	F(2,42) = 0.756, *p* = 0.476

Data are presented as mean ± standard deviation (SD). Abbreviations: *ST-MT* Soft-tissue–oriented manual therapy, *J-MT* Joint-oriented manual therapy, *SD* Standard deviation, *BMI* Body mass index

F: One-way analysis of variance (ANOVA) test statistic

**Table 2:** Changes in structural and functional outcomes following the intervention

Parameter	Group	Pre-intervention	Post-intervention	Change score ▲	F(2,42)	Group*time p	ηp^2^
Navicular drop (cm)	ST-MT	1.03 ± 0.07	0.71 ± 0.19	−0.33 ± 0.24	6.344	**0.004**	0.232
	J-MT	1.14 ± 0.28	0.85 ± 0.25	−0.29 ± 0.27			
	Control	1.06 ± 0.12	1.00 ± 0.24	−0.05 ± 0.16			
Foot Posture Index	ST-MT	7.27 ± 1.75	6.80 ± 1.82	−0.47 ± 0.52	6.500	**0.003**	0.236
	J-MT	8.53 ± 1.64	7.87 ± 2.00	−0.67 ± 0.72			
	Control	7.67 ± 1.80	7.80 ± 1.78	0.00 ± 0.38			
Weight bearing lunge test (cm)	ST-MT	9.47 ± 2.71	10.17 ± 2.81	0.50 ± 0.71	4.246	**0.021**	0.168
	J-MT	9.73 ± 2.60	10.97 ± 2.79	1.17 ± 0.88			
	Control	9.47 ± 1.92	9.87 ± 2.10	0.40 ± 0.63			
Y-Balance Test anterior reach distance (%)	ST-MT	59.73 ± 5.32	58.75 ± 4.86	−0.98 ± 3.21	7.335	**0.002**	0.259
	J-MT	55.18 ± 6.55	59.20 ± 4.36	4.02 ± 5.31			
	Control	57.22 ± 4.88	57.62 ± 4.42	0.40 ± 1.56			
Y-Balance Test posteromedial reach distance (%)	ST-MT	94.64 ± 11.62	91.98 ± 8.23	−2.67 ± 4.73	2.742	0.076	0.115
	J-MT	93.08 ± 8.91	94.31 ± 7.62	1.22 ± 5.57			
	Control	96.58 ± 6.30	97.02 ± 6.82	0.44 ± 4.02			
Y-Balance Test posterolateral reach distance (%)	ST-MT	92.91 ± 8.81	90.95 ± 7.89	−1.96 ± 5.41	0.496	0.613	0.023
	J-MT	94.02 ± 9.39	93.84 ± 9.08	−0.18 ± 4.25			
	Control	95.58 ± 7.63	94.42 ± 9.16	−1.16 ± 4.98			
Y-Balance Test composite score (%)	ST-MT	95.48 ± 6.03	93.35 ± 4.33	−2.12 ± 3.95	4.227	**0.021**	0.168
	J-MT	93.91 ± 6.15	95.88 ± 6.15	1.97 ± 4.37			
	Control	93.67 ± 4.88	93.57 ± 6.38	−0.10 ± 3.15			

Abbreviations *ST-MT* Soft-tissue–oriented manual therapy, *J-MT* Joint-oriented manual therapy, *SD* Standard deviation

*F* values represent group × time interaction effects from mixed ANOVA, ηp^2^: partial eta squared; *p* < 0.05 considered statistically significant

▲ Change score represents the difference between post-intervention and pre-intervention values

Bold values indicate statistically significant differences (*p* < 0.05)

### Foot morphology

Significant group × time interactions were found for both navicular drop (F = 6.344, *p* = 0.004, ηp^2^ = 0.232) and FPI (F = 6.500, *p* = 0.003, ηp^2^ = 0.236) (Table 2). Both ST-MT (MD: − 0.27 cm; 95% CI, − 0.47 to − 0.07; *p* = 0.006) and J-MT (MD: − 0.23 cm; 95% CI, − 0.43 to − 0.03; *p* = 0.020) demonstrated significantly greater reductions in ND compared with the control group. No significant difference was observed between ST-MT and J-MT (MD: − 0.04 cm; 95% CI, − 0.24 to 0.16; *p* = 0.880). Similarly, both ST-MT (MD: − 0.47; 95% CI, − 0.88 to − 0.06; *p* = 0.024) and J-MT (MD: − 0.67; 95% CI, − 1.20 to − 0.14; *p* = 0.012) demonstrated greater improvements in FPI compared with the control group. No significant difference was found between the intervention groups (MD: 0.20; 95% CI, − 0.37 to 0.77; *p* = 0.663) (Table 3).

**Table 3:** Between-group comparisons of changes in structural and functional outcomes

Parameter	Between group	MD and 95% CI	p
Navicular drop (cm)	ST-MT vs J-MT	−0.04 (−0.24 to 0.16)	0.880
	ST-MT vs C	−0.27 (−0.47 to −0.07)	**0.006**
	J-MT vs C	−0.23 (−0.43 to −0.03)	**0.020**
Foot posture index	ST-MT vs J-MT	0.20 (−0.37 to 0.77)	0.663
	ST-MT vs C	−0.47 (−0.88 to −0.06)	**0.024**
	J-MT vs C	−0.67 (−1.20 to −0.14)	**0.012**
Weight bearing lunge test (cm)	ST-MT vs J-MT	−0.67 (−1.33 to 0.00)	**0.048**
	ST-MT vs C	0.10 (−0.56 to 0.76)	0.929
	J-MT vs C	0.77 (0.10 to 1.43)	**0.020**
Y-Balance test anterior reach distance (%)	ST-MT vs J-MT	−5.00 (−9.01 to −0.99)	**0.013**
	ST-MT vs C	−1.37 (−3.71 to 0.96)	0.316
	J-MT vs C	3.62 (−0.05 to 7.30)	**0.050**
Y-Balance test posteromedial reach distance (%)	ST-MT vs J-MT	−3.89 (−8.16 to 0.38)	0.081
	ST-MT vs C	−3.11 (−7.39 to 1.16)	0.192
	J-MT vs C	0.78 (−3.49 to 5.05)	0.898
Y-Balance test posterolateral reach distance (%)	ST-MT vs J-MT	−1.78 (−6.13 to 2.57)	0.585
	ST-MT vs C	−0.80 (−5.15 to 3.55)	0.895
	J-MT vs C	0.98 (−3.37 to 5.33)	0.849
Y-Balance test composite score (%)	ST-MT vs J-MT	−4.09 (−7.51 to −0.67)	**0.016**
	ST-MT vs C	−2.03 (−5.45 to 1.39)	0.331
	J-MT vs C	2.07 (−1.35 to 5.49)	0.283

Abbreviations: *ST-MT* Soft-tissue–oriented manual therapy, *J-MT* Joint-oriented manual therapy, *C* Control, *MD* Mean differences, *CI* Confidence interval

Bonferroni-adjusted *p*-values are reported. Bold values indicate statistically significant differences (*p* < 0.05), MD values represent the mean difference between groups based on change scores

### Ankle dorsiflexion range of motion

A significant group × time interaction was observed for weight-bearing lunge test performance (F = 4.246, *p* = 0.021, ηp^2^ = 0.168) (Table 2). Post-hoc analyses demonstrated that the J-MT group showed significantly greater improvement compared with both the ST-MT group (MD: 0.67; 95% CI, 0.00 to 1.33; *p* = 0.048) and the control group (MD: 0.77; 95% CI, 0.10 to 1.43; *p* = 0.020). No significant difference was observed between the ST-MT and control groups (MD: 0.10; 95% CI, − 0.56 to 0.76; *p* = 0.929) (Tables 2 and 3).

### Dynamic balance

A significant group × time interaction was observed for Y-Balance Test anterior reach distance (F = 7.335, *p* = 0.002, ηp^2^ = 0.259) and composite score (F = 4.227, *p* = 0.021, ηp^2^ = 0.168) (Table 2). For anterior reach distance, the J-MT group showed greater improvement compared with ST-MT group (MD: 5.00; 95% CI, 0.99 to 9.01; *p* = 0.013). No significant differences were observed between either intervention group and the control group (*p* > 0.05). For the composite score, a significant difference was observed between the intervention groups, with the J-MT group demonstrating greater improvement compared with the ST-MT group (MD: 4.09; 95% CI, 0.67 to 7.51; *p* = 0.016). No statistically significant differences were observed between either intervention group and the control group (all *p* ≥ 0.05), including anterior reach distance (*p* = 0.050). No significant between-group differences were observed for posteromedial reach distance (F = 2.742, *p* = 0.076, ηp^2^ = 0.115) or posterolateral reach distance (F = 0.496, *p* = 0.613, ηp^2^ = 0.023) (Tables 2 and 3).

### Passive mechanical properties of soft tissues

Significant group × time interactions were identified for soleus tone (F = 4.247, *p* = 0.021, ηp^2^ = 0.175) and soleus elasticity (F = 5.940, *p* = 0.005, ηp^2^ = 0.225). Post-hoc analyses revealed that soleus tone decreased significantly in the ST-MT group compared with the control group (MD: − 1.97; 95% CI, − 3.68 to − 0.26; *p* = 0.020). For soleus elasticity, both ST-MT (MD: − 0.25; 95% CI, − 0.43 to − 0.07; *p* = 0.005) and J-MT (MD: − 0.19; 95% CI, − 0.37 to − 0.01; *p* = 0.042) demonstrated significant improvements in elasticity, as reflected by reduced logarithmic decrement values compared with the control group. (Tables 4 and 5).

**Table 4:** Changes in passive mechanical muscle properties following the intervention

Muscle	Property	Group	Pre-intervention	Post-intervention	Change score ▲	F(2,42)	p	ηp^2^
Medial gastrocnemius	Tone	ST-MT	14.64 ± 2.99	13.95 ± 2.39	−0.68 ± 1.15	1.524	0.230	0.069
		J-MT	13.72 ± 2.24	13.72 ± 2.20	−0.01 ± 0.85			
		Control	14.76 ± 2.47	14.56 ± 2.48	−0.20 ± 1.19			
	Stiffness	ST-MT	263.53 ± 78.00	249.44 ± 61.86	−14.09 ± 28.17	0.328	0.722	0.015
		J-MT	266.31 ± 84.62	254.22 ± 67.65	−12.09 ± 36.07			
		Control	270.51 ± 78.26	265.75 ± 82.02	−4.76 ± 34.86			
	Elasticity	ST-MT	1.37 ± 0.20	1.40 ± 0.18	0.03 ± 0.25	0.413	0.664	0.019
		J-MT	1.46 ± 0.22	1.42 ± 0.16	−0.03 ± 0.21			
		Control	1.28 ± 0.19	1.24 ± 0.33	−0.03 ± 0.22			
Lateral gastrocnemius	Tone	ST-MT	14.98 ± 2.97	14.35 ± 2.87	−0.62 ± 1.72	0.815	0.449	0.037
		J-MT	14.20 ± 2.66	13.90 ± 2.42	−0.30 ± 0.98			
		Control	16.11 ± 3.06	16.01 ± 3.10	−0.46 ± 1.05			
	Stiffness	ST-MT	274.05 ± 104.72	260.00 ± 92.74	−14.05 ± 48.64	0.100	0.905	0.005
		J-MT	259.82 ± 72.38	246.66 ± 59.89	−13.15 ± 34.69			
		Control	296.60 ± 76.88	288.38 ± 69.12	−8.22 ± 29.24			
	Elasticity	ST-MT	1.32 ± 0.34	1.20 ± 0.13	−0.13 ± 0.29	1.288	0.286	0.058
		J-MT	1.22 ± 0.14	1.22 ± 0.18	0.00 ± 0.12			
		Control	1.24 ± 0.19	1.19 ± 0.15	−0.05 ± 0.19			
Soleus	Tone	ST-MT	17.76 ± 2.89	16.05 ± 2.61	−1.71 ± 2.23	4.247	**0.021**	0.175
		J-MT	17.52 ± 3.49	16.33 ± 3.03	−1.19 ± 1.57			
		Control	17.15 ± 2.68	17.47 ± 3.28	0.26 ± 1.89			
	Stiffness	ST-MT	340.09 ± 67.92	312.44 ± 60.23	−27.65 ± 50.17	2.292	0.114	0.103
		J-MT	331.98 ± 83.93	314.57 ± 72.84	−17.40 ± 31.42			
		Control	329.38 ± 69.38	335.24 ± 69.56	4.22 ± 43.35			
	Elasticity	ST-MT	1.83 ± 0.23	1.72 ± 0.20	−0.11 ± 0.18	5.940	**0.005**	0.225
		J-MT	1.89 ± 0.29	1.84 ± 0.20	−0.05 ± 0.21			
		Control	1.68 ± 0.28	1.81 ± 0.29	0.14 ± 0.21			
Tibialis anterior	Tone	ST-MT	23.76 ± 5.30	22.58 ± 5.73	−1.18 ± 3.31	0.414	0.664	0.019
		J-MT	22.32 ± 4.35	21.96 ± 3.72	−0.36 ± 2.84			
		Control	23.18 ± 4.48	22.90 ± 5.45	−0.29 ± 2.73			
	Stiffness	ST-MT	534.91 ± 185.78	482.02 ± 171.58	−52.89 ± 63.65	3.229	**0.050**	0.133
		J-MT	494.04 ± 125.05	482.18 ± 98.74	−11.87 ± 59.21			
		Control	501.91 ± 129.29	503.15 ± 146.46	1.24 ± 59.63			
	Elasticity	ST-MT	1.45 ± 0.19	1.45 ± 0.18	0.00 ± 0.16	0.029	0.971	0.001
		J-MT	1.40 ± 0.29	1.41 ± 0.27	0.01 ± 0.21			
		Control	1.50 ± 0.25	1.49 ± 0.38	−0.01 ± 0.34			
Achilles tendon	Tone	ST-MT	30.51 ± 4.87	30.46 ± 5.13	−0.06 ± 4.10	1.837	0.172	0.080
		J-MT	29.62 ± 2.87	27.53 ± 3.99	−2.09 ± 3.94			
		Control	29.53 ± 3.42	30.62 ± 7.30	1.10 ± 5.59			
	Stiffness	ST-MT	915.16 ± 243.38	898.69 ± 255.71	−16.47 ± 174.62	1.329	0.276	0.060
		J-MT	864.38 ± 182.90	744.62 ± 156.88	−119.76 ± 202.50			
		Control	877.65 ± 215.88	864.38 ± 377.42	−13.27 ± 229.63			
	Elasticity	ST-MT	1.18 ± 0.18	1.12 ± 0.12	−0.06 ± 0.18	1.279	0.289	0.057
		J-MT	1.13 ± 0.15	1.19 ± 0.19	0.06 ± 0.14			
		Control	1.08 ± 0.11	0.99 ± 0.36	−0.09 ± 0.41			
Plantar fascia	Tone	ST-MT	25.61 ± 3.24	25.38 ± 3.45	−0.23 ± 1.51	0.599	0.554	0.028
		J-MT	26.36 ± 2.67	27.03 ± 3.93	0.67 ± 3.09			
		Control	25.25 ± 2.70	25.31 ± 2.84	0.07 ± 1.97			
	Stiffness	ST-MT	603.33 ± 97.66	603.71 ± 99.90	0.38 ± 41.24	1.363	0.267	0.061
		J-MT	640.82 ± 68.13	639.62 ± 84.29	−1.20 ± 59.94			
		Control	653.20 ± 93.72	681.11 ± 94.35	27.91 ± 59.64			
	Elasticity	ST-MT	1.54 ± 0.18	1.61 ± 0.25	0.07 ± 0.18	1.277	0.290	0.057
		J-MT	1.60 ± 0.22	1.61 ± 0.26	0.02 ± 0.17			
		Control	1.73 ± 0.26	1.69 ± 0.28	−0.03 ± 0.16			

Abbreviations: *ST-MT* Soft-tissue–oriented manual therapy, *J-MT* Joint-oriented manual therapy, *C* Control, SD = standard deviation

*F* values represent group × time interaction effects from mixed ANOVA, ηp^2^: partial eta squared; *p* < 0.05 considered statistically significant

▲ Change score represents the difference between post-intervention and pre-intervention values

Bold values indicate statistically significant differences (*p* < 0.05)

**Table 5:** Between-group comparisons of changes in passive mechanical properties

Muscle	Property	Between group	MD and 95% CI	p
Medial gastrocnemius	Tone	ST-MT vs J-MT	−0.68 (−1.66 to 0.30)	0.222
		ST-MT vs C	−0.48 (−1.44 to 0.48)	0.450
		J-MT vs C	0.20 (−0.78 to 1.18)	0.875
	Stiffness	ST-MT vs J-MT	−2.00 (−31.46 to 27.47)	0.985
		ST-MT vs C	−9.33 (−38.80 to 20.13)	0.724
		J-MT vs C	−7.34 (−36.80 to 22.13)	0.818
	Elasticity	ST-MT vs J-MT	0.06 (−0.14 to 0.27)	0.723
		ST-MT vs C	0.07 (−0.14 to 0.27)	0.704
		J-MT vs C	0.00 (−0.20 to 0.20)	0.999
Lateral gastrocnemius	Tone	ST-MT vs J-MT	−0.32 (−1.47 to 0.82)	0.774
		ST-MT vs C	−0.16 (−1.31 to 0.99)	0.939
		J-MT vs C	0.16 (−0.98 to 1.31)	0.936
	Stiffness	ST-MT vs J-MT	−0.89 (−34.96 to 33.18)	0.998
		ST-MT vs C	−5.82 (−39.89 to 28.24)	0.909
		J-MT vs C	−4.93 (−39.00 to 29.14)	0.934
	Elasticity	ST-MT vs J-MT	−0.12 (−0.31 to 0.07)	0.260
		ST-MT vs C	−0.08 (−0.26 to 0.11)	0.601
		J-MT vs C	0.05 (−0.14 to 0.24)	0.807
Soleus	Tone	ST-MT vs J-MT	−0.52 (−2.26 to 1.22)	0.745
		ST-MT vs C	−1.97 (−3.68 to −0.26)	**0.020**
		J-MT vs C	−1.45 (−3.19 to 0.29)	0.120
	Stiffness	ST-MT vs J-MT	−10.24 (−48.73 to 28.25)	0.795
		ST-MT vs C	−31.87 (−69.69 to 5.95)	0.113
		J-MT vs C	−21.63 (−60.11 to 16.86)	0.368
	Elasticity	ST-MT vs J-MT	−0.06 (−0.24 to 0.12)	0.695
		ST-MT vs C	−0.25 (−0.43 to −0.07)	**0.005**
		J-MT vs C	−0.19 (−0.37 to −0.01)	**0.042**
Tibialis anterior	Tone	ST-MT vs J-MT	−0.82 (−3.46 to 1.82)	0.734
		ST-MT vs C	−0.89 (−3.53 to 1.75)	0.694
		J-MT vs C	−0.07 (−2.71 to 2.57)	0.998
	Stiffness	ST-MT vs J-MT	−41.02 (−95.02 to 12.97)	0.167
		ST-MT vs C	−54.13 (−108.13 to −0.14)	**0.049**
		J-MT vs C	−13.11 (−67.10 to 40.88)	0.826
	Elasticity	ST-MT vs J-MT	−0.01 (−0.23 to 0.21)	0.997
		ST-MT vs C	0.01 (−0.20 to 0.23)	0.986
		J-MT vs C	0.02 (−0.20 to 0.24)	0.970
Achilles tendon	Tone	ST-MT vs J-MT	2.03 (−2.05 to 6.12)	0.455
		ST-MT vs C	−1.15 (−5.24 to 2.93)	0.774
		J-MT vs C	−3.18 (−7.27 to 0.90)	0.153
	Stiffness	ST-MT vs J-MT	103.29 (−77.23 to 283.81)	0.355
		ST-MT vs C	−3.20 (−183.72 to 177.32)	0.999
		J-MT vs C	−106.49 (−287.01 to 74.03)	0.333
	Elasticity	ST-MT vs J-MT	−0.12 (−0.36 to 0.12)	0.469
		ST-MT vs C	0.03 (−0.21 to 0.27)	0.939
		J-MT vs C	0.15 (−0.09 to 0.39)	0.291
Plantar fascia	Tone	ST-MT vs J-MT	−0.90 (−2.93 to 1.13)	0.536
		ST-MT vs C	−0.29 (−2.32 to 1.74)	0.935
		J-MT vs C	0.60 (−1.43 to 2.64)	0.751
	Stiffness	ST-MT vs J-MT	1.58 (−46.60 to 49.76)	0.997
		ST-MT vs C	−27.53 (−75.71 to 20.65)	0.356
		J-MT vs C	−29.11 (−77.29 to 19.07)	0.316
	Elasticity	ST-MT vs J-MT	0.05 (−0.10 to 0.20)	0.676
		ST-MT vs C	0.10 (−0.05 to 0.25)	0.258
		J-MT vs C	0.05 (−0.10 to 0.20)	0.736

Abbreviations: *ST-MT* Soft-tissue–oriented manual therapy, *J-MT* Joint-oriented manual therapy, *C* Control, *MD* Mean differences, CI = confidence interval

Bonferroni-adjusted p-values are reported. Bold values indicate statistically significant differences (*p* < 0.05), MD values represent the mean difference between groups based on change scores

For the tibialis anterior, a significant group × time interaction was detected for stiffness (F = 3.229, *p* = 0.050, ηp^2^ = 0.133). Post-hoc comparisons showed that ST-MT (MD: − 54.13; 95% CI, − 108.13 to − 0.14; *p* = 0.049) demonstrated greater reductions compared with the control group. No significant interactions were observed for tibialis anterior tone or elasticity (*p* > 0.05).

No significant group × time interactions were observed for tone, stiffness, or elasticity of the medial and lateral gastrocnemius muscles, plantar fascia, or Achilles tendon (all *p* > 0.05) (Table 4).

## Discussion

This randomized controlled trial compared the immediate effects of joint-oriented manual therapy and soft-tissue-oriented manual therapy in physically active individuals with flexible pes planus. Both interventions were associated with immediate improvements in foot morphology compared with baseline, as reflected by changes in medial longitudinal arch-related measures. However, the two modalities exhibited distinct functional and mechanical profiles. J-MT demonstrated greater improvements compared with ST-MT in ankle dorsiflexion range of motion, while improvements in dynamic balance outcomes were limited and not consistently different from the control group. Conversely, ST-MT was particularly effective in modulating the mechanical properties of the soleus muscle, particularly tone and elasticity. It was also associated with a reduction in tibialis anterior stiffness compared with the control group. No significant changes were observed in plantar fascia or Achilles tendon mechanical properties following either intervention.

### Morphological changes in foot alignment

FPP is characterized by medial arch collapse and excessive pronation during weight-bearing, often associated with altered lower-limb biomechanics and soft-tissue adaptations [1, 2]. In the present study, both manual therapy approaches significantly reduced ND and FPI scores compared with the control group. The magnitude of change (approximately 0.24–0.28 cm in ND) suggests an immediate, clinically observable change in medial longitudinal arch-related measures following a single session.

These findings are consistent with prior evidence suggesting that both joint mobilization and myofascial techniques may acutely influence foot posture and overall lower-limb alignment [11, 29]. The underlying mechanism may involve reduced passive resistance within the soleus muscle, potentially contributing to improved load distribution across the medial arch [30]. Additionally, restoration of subtalar and midtarsal joint congruence through J-MT may contribute to improved medial column alignment through improved arthrokinematics [30, 31]. Although only immediate effects were examined, the observed short-term structural changes suggest that manual therapy may transiently modify arch mechanics in FPP, which may be clinically relevant prior to dynamic or sport-specific tasks.

### Changes in dorsiflexion range of motion

Only the J-MT group demonstrated significantly greater improvements in dorsiflexion compared with both ST-MT (MD: 0.67 cm) and the control group (MD: 0.77 cm), suggesting that joint-oriented mobilization may provide a mechanical advantage in restoring talocrural mobility. This supports earlier evidence that joint mobilization targeting posterior talar glide has been shown to improve dorsiflexion range of motion and functional performance in ankle-related pathologies [32–35]. Given that anterior reach performance and functional squat depth are closely associated with talocrural mobility [36], even modest acute increases in weight-bearing dorsiflexion may contribute to improved dynamic postural control.

In contrast, ST-MT did not demonstrate significant differences compared with the control group in between-group comparisons. This suggests that changes in soft-tissue mechanical properties alone may be insufficient to overcome articular restrictions during a single treatment session. Repeated myofascial interventions may be required to produce comparable mobility gains [12].

### Dynamic balance

Improvements in selected dynamic balance parameters were observed primarily in the J-MT group, particularly in anterior reach distance and composite score. The J-MT group demonstrated a significantly greater improvement in anterior reach distance than the ST-MT group (MD: 5.00), whereas the comparison with the control group did not reach statistical significance (*p* = 0.050). While ST-MT primarily targets the viscoelastic properties of soft tissues, J-MT directly addresses the joint capsule and accessory glides of the talus. This may facilitate the posterior talar glide required to accommodate increased ankle dorsiflexion during dynamic weight-bearing tasks [36], which is a key determinant of anterior reach performance. Consequently, these findings suggest that articular mobility, rather than isolated modulation of soft-tissue mechanical properties, may contribute to improvements in certain dynamic balance parameters; however, these findings should be interpreted cautiously given the lack of consistent differences compared with the control group in individuals with flexible pes planus.

### Modulation of muscle mechanical properties

Our results revealed that J-MT and ST-MT appear to influence different aspects of the mechanical properties of lower limb tissues, likely reflecting their different physiological mechanisms. To the best of our knowledge, no previous studies have directly compared J-MT and ST-MT on the mechanical properties of muscles in individuals with FPP. The ST-MT intervention was effective in modulating specific properties of the soleus muscle, particularly by reducing muscle tone, while both ST-MT and J-MT improved elasticity (i.e., reduced logarithmic decrement values) compared with the control group. These findings are consistent with studies reporting immediate decreases in muscle tone following myofascial release [26, 37, 38]. The broad application area in the present study may have contributed to broader changes in posterior compartment muscle properties compared with protocols limited to localized treatment [37–39]. J-MT, however, did not demonstrate significant effects on stiffness parameters across the assessed tissues. In contrast to Tan et al. [37], our results did not demonstrate significant changes in structural stiffness of deep supportive tissues. This difference likely reflects population characteristics, as FPP involves chronic biomechanical adaptations rather than scar-related stiffness, suggesting that restoring talocrural arthrokinematics through J-MT alone may not be sufficient to acutely modify stiffness parameters. In contrast, soft-tissue–oriented techniques may have a more direct influence on muscle mechanical properties.

The soleus muscle exhibited measurable responses to both interventions. Both groups demonstrated improvements in elasticity compared with the control group, while a significant reduction in tone was observed only in the ST-MT group. These effects align with the distinct mechanical targets of the two techniques, where both ST-MT and J-MT may influence viscoelastic properties of the posterior compartment. While no significant between-group changes were observed in stiffness, the improvements in elasticity and, to a lesser extent, tone suggest that both soft-tissue–oriented manual therapy and joint-oriented manual therapy may acutely influence specific passive mechanical properties of the soleus muscle in individuals with FPP.

For the tibialis anterior, a significant reduction in stiffness was observed only in the ST-MT group compared with the control group, whereas no significant differences were found for J-MT. This finding may be attributed to the direct mechanical effects of soft-tissue–oriented techniques on superficial muscle structures, potentially leading to acute reductions in tissue resistance.

In contrast, no significant between-group differences were observed in the mechanical properties of the Achilles tendon or plantar fascia. These findings suggest that a single session of either intervention may be insufficient to induce measurable changes in denser or more structurally complex tissues. Accordingly, repeated or longer-duration interventions may be required to achieve meaningful modifications in these tissues in individuals with FPP.

### Clinical ımplications and limitations

From a clinical perspective, the results support a differentiated treatment approach in FPP. When the primary goal is to enhance dorsiflexion and dynamic balance performance, joint-oriented mobilization may provide greater improvements in certain outcomes, particularly ankle dorsiflexion, compared with soft-tissue techniques. Conversely, when elevated muscle tone predominates, ST-MT may be preferentially selected. Importantly, both interventions produced distinct mechanical adaptations, suggesting that a combined approach may be beneficial in addressing both articular and soft-tissue components within the same session. However, these findings reflect only the immediate effects of a single treatment session and should therefore be interpreted cautiously until longer-term studies confirm the persistence and clinical relevance of these changes.

Several limitations should be considered. The study evaluated only the immediate effects of a single session; thus, the persistence and cumulative benefits of repeated treatments remain unknown. Second, mechanical properties were assessed in a relaxed, non-weight-bearing position; while this approach ensures reproducibility, it may not fully represent functional conditions. In addition, the absence of a sham or placebo manual contact condition limits the interpretation of intervention-specific effects beyond time-related changes. Finally, the sample included only physically active young adults with asymptomatic flexible pes planus, which may limit the generalizability of the findings to symptomatic individuals, older adults, sedentary populations, or individuals with more severe deformities and pain-related functional limitations.

## Conclusion

This trial provides insight into the immediate effects of different manual therapy strategies on lower limb mechanical behavior in individuals with flexible pes planus. Both joint- and soft-tissue–oriented manual therapies resulted in immediate changes in selected structural, functional, and mechanical parameters.

Joint-oriented mobilization was associated with greater improvements in ankle dorsiflexion and selected dynamic balance parameters compared with ST-MT; however, dynamic balance findings should be interpreted cautiously given the limited between-group differences compared with the control group. Soft-tissue–oriented manual therapy was particularly effective in reducing soleus muscle tone and improving elasticity, and also led to a reduction in tibialis anterior stiffness.

No significant changes were observed in the mechanical properties of the Achilles tendon or plantar fascia following either intervention. These findings indicate that the effects of manual therapy are parameter- and tissue-specific, highlighting the potential value of tailoring interventions according to the targeted functional and mechanical outcomes.

## Supplementary Information

Below is the link to the electronic supplementary material.

Supplementary file1 (DOCX 420 KB) Detailed description of joint-oriented and soft tissue–oriented manual therapy protocols, including application procedures, duration, and targeted structures

## Data Availability

The datasets generated and/or analysed during the current study are not publicly available due to privacy and ethical restrictions but are available from the corresponding author on reasonable request.
