# Supplementary material for: Immediate effects of joint- versus soft-tissue-oriented manual therapy on lower-limb function and muscle mechanics in individuals with flexible pes planus: a randomized controlled trial
Source: Ir J Med Sci. 2026 May 26;195(5):2811–22. doi: 10.1007/s11845-026-04464-2 (PMC13739974; doi:10.1007/s11845-026-04464-2)
Supplement: Supplementary file 1 [file 11845_2026_4464_MOESM1_ESM.docx]

**Supplementary Information (Online Resource 1)**

**Article Title:** Immediate Effects of Joint- Versus Soft-Tissue-Oriented Manual Therapy on Lower-Limb Function and Muscle Mechanics in Individuals with Flexible Pes Planus: A Randomized Controlled Trial

**Journal:** Irish Journal of Medical Science

**Authors:** Bensu Sogut-Kazan, Ezgi Unuvar-Yuksel, Hande Guney-Deniz

**Corresponding Author:**

Hande GÜNEY-DENİZ, PT, PhD, Prof. Dr
**Address:** Hacettepe University, Faculty of Physical Therapy and Rehabilitation, Department of Musculoskeletal Physiotherapy and Rehabilitation, 06100, Ankara, Turkiye.

**E-mail:** hande.guney@hacettepe.edu.tr

**Intervention Protocols**

The specific application techniques were as follows:

**Soft Tissue-Oriented Manual Therapy**

**Figure S1**. Application of soft tissue–oriented manual therapy to the gastrocnemius muscle.

|  | Participants were positioned prone. The physiotherapist's elbow was placed at 90° flexion, starting from the participant’s Achilles tendon and moving proximally while maintaining application pressure. Simultaneously, the participant was asked to actively dorsiflex their foot. This technique was performed for 15 repetitions. |
| --- | --- |

**Figure S2.** Application of soft tissue–oriented manual therapy to the soleus muscle.

|  | Participants were asked to lie prone, and their leg was positioned in 10° to 15° flexion using a supportive pillow. The physiotherapist's elbow was positioned at 90° over the Achilles tendon, and pressure was gradually applied towards the fascial layer located between the soleus and gastrocnemius muscles. The pressure was tracked from distal to proximal, while the participant was requested to dorsiflex their foot. This technique was performed for 15 repetitions |
| --- | --- |

**Figure S3.** Application of soft tissue–oriented manual therapy to the Achilles tendon.

|  | While the participant was lying prone, the physiotherapist contacted the medial and lateral borders of the calcaneus with their index, middle, and ring fingers, and maintained the pressure proximally. During this, the participant was asked to perform three repetitions of dorsiflexion. This technique was performed for 15 repetitions. |
| --- | --- |

**Figure S4.** Application of soft tissue–oriented manual therapy to the plantar fascia.

|  | - The physiotherapist formed a fist and placed the dorsal surface of their phalanges beneath the calcaneus, following the fascia with pressure toward the participant's toes. Participants were asked to move their toes in the flexion-extension direction simultaneously. This technique was performed for 15 repetitions |
| --- | --- |

**Joint-Oriented Manual Therapy**

**Figure S5.** Non–weight-bearing antero-posterior talocrural joint mobilization.

|  | The participant was positioned prone, and the leg to be treated was placed in 90° knee flexion. The physiotherapist stabilized the tibia and fibula at the level of the malleoli with one hand, while grasping the midfoot with the other hand to apply antero-posterior (AP) mobilization. Arrows indicate the direction of force application. |
| --- | --- |

**Figure S6.** Weight-bearing antero-posterior talocrural joint mobilization using a mobilization belt.

|  | The participant placed the foot to be treated on a chair positioned in front of them and flexed the knee. The physiotherapist stood opposite the participant and stabilized the foot with one hand. A mobilization belt was placed on the distal posterior calf region, with a thin cushion or towel placed underneath the belt to prevent pain. The belt was attached to the physiotherapist's pelvis. In this position, as the participant performed active dorsiflexion, the physiotherapist applied an antero-posterior mobilization by leaning backward with their trunk. |
| --- | --- |

**Figure S7.** Subtalar joint eversion–inversion mobilization.

|  | While the participant was lying prone, the leg to be treated was placed in 90° knee flexion. The physiotherapist stood beside the participant, grasped the calcaneus, and applied eversion and inversion mobilization by holding the heel while the sole of the foot was kept parallel to the ceiling. Arrows indicate the direction of force application. |
| --- | --- |

**Figure S8.** Midtarsal joint pronation–supination mobilization.

|  | While the participant was lying prone, the leg to be treated was placed in 90° knee flexion. The physiotherapist stood beside the participant, stabilized the calcaneus and talus with one hand, and applied rotational mobilization to the midtarsal joint in the pronation and supination directions with the other hand. Arrows indicate the direction of force application. |
| --- | --- |
